# Neuropsychiatric Symptoms as The First Manifestation of Juvenile Systemic Lupus Erythematosus: A Complicated Case with Klinefelter’s Syndrome

**Published:** 2014

**Authors:** Reza SHIARI, Mehrnoush HASSASE YEGANE, Shirin FARIVAR, Vadood JAVADI PARVANEH, Seyed Alireza MIRJAVADI

**Affiliations:** 1 Department of Paediatric Rheumatology, Shahid Behshti University of Medical Sciences, Mofid Children’s Hospital, Tehran, Iran.; 2 Department of Genetics, Faculty of Biological Science, Shahid Beheshti University, GC, Tehran, Iran.

**Keywords:** Juvenile Systemic Lupus Erythematosus, Klinefelter’s syndrome, Cognitive Dysfunction, Neuropsychiatric

## Abstract

Systemic Lupus Erythematosus (SLE) is an autoimmune, multisystem disorder with various manifestations. There are limited reports on the neuropsychiatric findings as the first manifestation of SLE in children. Herein, we report a 14-year-old Iranian boy with a two-year history of cognitive dysfunction and behavioural problems as well as a recent history of epistaxis. The patient workup ended with a diagnosis of Klinefelter’s syndrome associated with juvenile SLE. Patients with Klinefelter’s syndrome may exhibit behavioural problems and psychological disease. These psychiatric disorders could be complicated with lupus in children. In fact, psychiatric symptoms may occur as the first manifestation of juvenile SLE. Specially, if accompanied with Klinefelter’s syndrome. We suggest the diagnosis of SLE must be considered in all children with neuropsychiatric manifestations.

## Introduction

Systemic Lupus Erythematosus (SLE) is an autoimmune, inflammatory disorder affecting multiple organ systems (1). The nervous system is affected in more than half of all patients during the course of the disease, both in children and adults, and its involvement is associated with a worse prognosis and more cumulative damage (2, 3). Neuropsychiatric Lupus (NPSLE) manifestations can occur in the absence of either serologic activity or other systemic disease manifestations (4). Patients with Klinefelter’s syndrome (KFS) show a higher percent of SLE diseases than the normal population (5). On the other hand, psychiatric disorders involving anxiety, depression, neurosis, and psychosis are more common in this group than in the general population (6). SLE may be the first presenting symptom of Klinefelter’s syndrome and lead to a diagnosis of the disease (7). Herein, we present a case with Klinefelter’s syndrome and psychiatric disorders. In due course, this patient exhibited clinical features of SLE and was diagnosed with a case of neuropsychiatric lupus associated with KFS.

## Case presentation

A fourteen year-old Iranian boy who suffered from headaches and lethargy for two years was admitted to Mofid Children’s Hospital. During the two years, he had demonstrated aggressive behaviour, a loss of school performance, stuttering of nails, and eyelash and eyebrow ablation. Last year, he was admitted to a psychiatric centre because of behavioural, memory, and psychiatric problems. He received various treatments, including speech therapy, physical therapy, and psychotherapy. None of these measures was helpful and he continued the abnormal behaviour. For the last month, he suffered from epistaxis and easy bruising, for which, he was again referred to Mofid Children’s Hospital. During admission, he showed photosensitivity and photophobia. On physical examination, his eyebrows and eyelashes were absent and he had no nails on fingers. He had oral ulcers, scant facial and body hair, multiple ecchymoses, and petechiae on his body. The laboratory findings revealed thrombocytopenia with positive titres of antinuclear antibodies (ANA), anti-double stranded DNA antibody, antiphospholipid antibody, and low titres of C3 and C4. There was neither anaemia nor leukopenia. The results of direct and indirect Coombs tests were negative. His bone marrow aspiration and coagulation profile were normal. Results of other blood tests were within normal limits or negative, including lupus anticoagulant, ß2-glycoprotein, anticardiolipin IgG and IgM Abs, anti-RO, anti-LA, anti-SM Ab, P & C-ANCA, VDRL, TSH, FT4, anti-T microsomal, antithyroglobulin, cryoglobulins, AntiHIV antibody hepatitis C antibodies, hepatitis B antigen, protein C activity, and protein S free. He had no evidence of uveitis or vasculitis and his brain MRI was normal. 

**Fig 1:**
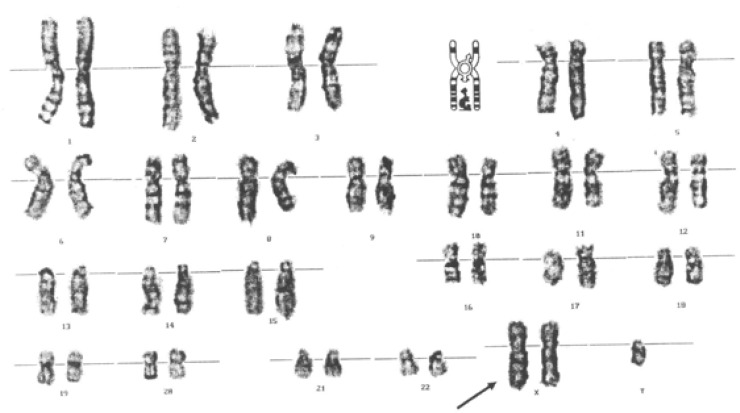
10 metaphase spreads from a bone marrow sample and 15 metaphase spreads from peripheral blood sample

His Karyotyping revealed 47 XXY, which is diagnostic for Klinefelter’s syndrome (Fig. 1).

The patient fulfilled the ACR classification Criteria (revised 1997) for juvenile SLE (8). Therefore, he was treated with parenteral corticosteroids and cyclophosphamide pulse therapy. After two weeks of treatment, his neuropsychiatric symptoms and thrombocytopenia began to normalize.

## Discussion

SLE is a chronic autoimmune disease characterized by multisystemic involvement and diverse manifestations (9). Central nervous system involvement remains a major cause of morbidity and mortality in these types of patients (10). The overall prevalence of major NPSLE varies among series. It ranges widely between 22% and 95% in children to reflect variable diagnostic criteria and the difficulty in defining psychiatric abnormalities as well as differences in selection of patients for study (1, 7, 13). ACR has developed a standardized nomenclature system that provides case definitions for neuropsychiatric syndromes in SLE (10, 12). In the only prospective study of NPSLE in children, nervous system manifestations were more common over a 6-year study period than glomerulonephritis (95% versus 55%) (14). Headaches occurred in 55%, were studied based on GTG technique at 400-450 band resolution, revealing 47-XXY pattern. mood disorders in 57%, cognitive dysfunction in 55%, seizure disorders in 51%, acute confusional disorders in 35%, peripheral nervous system impairment in 15%, psychosis in 12%, and stroke in 12% (14). Another retrospective study of NPSLE in children found NPSLE manifestations to be common and occurred early in the course of the disease. However, these manifestations were not necessarily associated with disease activity outside the nervous system (15). 

A direct pathogenic role of autoantibodies in NPSLE through induction of neural dysfunction, vasculopathy, and coagulopathy has been suggested (16). Karyotyping of our patient showed that he was 47-XXY. On the other hand, humoral and cellular immunity are enhanced in Klinefelter’s syndrome, because of testosterone deficiency and increased levels of estradiol that enhance autoantibody production (17).

It may be proposed that this is the possible reason of neuropsychiatric symptoms in this patient. 

The presence of antiphospholipid antibodies was seen in 70% of children and the association of these antibodies with Klinefelter’s syndrome is incorrigible (16, 17). Antibodies to phospholipids and phospholipidbinding proteins like β2GP-1 can cause thrombosis and other antibody-mediated clinical manifestations, such as stroke (1, 16). However, much attention is currently directed at the potential role of anti-glutamate receptor antibodies in cognitive dysfunction and psychiatric disease in patients with SLE (1, 16, 18). Our patient showed high levels of anti-phospholipid antibodies (IgM = 35 u/dl and IgG = 56 u/dl) that may explain the headaches.

There are some reports of psychiatric manifestations from SLE before systemic presentation of it (19-20). Our patient had shown some psychiatric manifestations long before the appearance of the classic systemic presentation of SLE. However, the classic psychiatric treatments were not effective to remove his symptoms. His karyotyping was also in favour of Klinefelter’s syndrome. Therefore, our patient’s neuropsychiatric manifestations occurred because of Klinefelter’s syndrome that was complicated by Lupus.

**In conclusion, **in paediatric patients with psychiatric disorders, such as psychosis, mood disorders, personality changes, or behavioural disorders, especially in those who do not respond well to the classic psychiatric treatments, a diagnosis of SLE must be strongly considered.
